# Evaluation of Nurses’ Practice Environment in Cardiovascular Hospitals: Nurses’ Perceptions

**DOI:** 10.1155/jonm/4507634

**Published:** 2026-05-08

**Authors:** Ali Hussein Alek Al-Ganmi, Tahseen Rajab Mohammed, Ayad Majid Mousa, Haider Mohammed Majeed, Abdulellah M. Alsolais

**Affiliations:** ^1^ Department of Adult Nursing, College of Nursing, University of Baghdad, Baghdad, Iraq, uobaghdad.edu.iq; ^2^ College of Applied Medical Sciences, Shaqra University, Shaqra, Saudi Arabia, su.edu.sa

**Keywords:** cardiovascular hospitals, evaluate, nurses, perception, practice environment, XXX

## Abstract

**Background:**

An attractive practice environment for nurses is essential in cardiac hospitals; however, limited studies are known about practice environment in this setting. This study aimed to evaluate nurses’ perceptions of the practice environment in **XXX** cardiac hospitals. Additionally, this study seeks to identify nurse‐related factors that influence nurses’ nursing practices in these hospitals.

**Methods:**

A cross‐sectional study utilizing a self‐report questionnaire was conducted among a convenience sample of 115 nurses working in cardiac hospitals. Nurses’ perceptions of the practice environment in cardiac hospitals were explored via the Nursing Work Index. A descriptive analysis was performed in the form of categorical variables presented as frequencies and percentages, and the continuous variables were presented as the means and standard deviations. Multiple linear regression was performed to analyze the relationship between sociodemographic factors and the level of environmental practice. The level of significance was set at a *p* value = (*p* ≤ 0.05).

**Results:**

The findings indicate an overall favorable practice environment, with 56% of the nurses reporting a good level of practice in the cardiac setting. Cardiac nurses held a positive perception of their nursing practice in cardiac care settings. Overall, the participants responded well, with a total mean score of (M ± SD = 3.38 ± 0.02) for their perceptions of leadership, staff support, nursing supervisory skills, participation in hospital committees, staffing and resource adequacy and collegial nurse‒physician relations. Nursing qualifications, years of experience and participation in nursing work management‐related training courses were predictors of cardiac nurses’ perceptions of the practice environment (*p* value = *p* ≤ 0.014).

**Conclusion:**

Survey nurses’ perceptions of the practice environment in cardiac hospitals provide insight into nurses’ actual readiness for providing high‐quality care and hence positive patient health outcomes. Cardiac nurses should be involved in decision‐making authority and advocate for the workplace to better support their independence, all to improve the nursing practice environment in cardiovascular hospitals.

## 1. Introduction

A high level of nursing care necessitates a sufficient number of specialized skilled nurses in cardiac hospitals, who are considered the major health work force [1]. Cardiac nurses are admired for their tenacity, stoicism, and ability to work under pressure and limited resources [2]. However, cardiac nurses endure higher levels of burnout and posttraumatic stress, which is a major concern globally compared with other healthcare workers because of job dissatisfaction, excessive job demands, and insufficient empowerment [3]. Retaining qualified nurses in cardiac care settings might be impacted by a variety of individual and professional factors [4]. Cardiac nursing poses various practical challenges for nurses in the clinical setting, including elevated patient acuity and deterioration, intricate technology, substantial workload, and emotional and psychological distress. In particular, novel nurses are less likely to work in environments with high practical demands, such as critical care and cardiac care [5]. The likelihood of burnout, work team conflict, and quitting jobs among cardiac nurses due to the impact of the practice environment might directly affect their work‒life balance and, consequently, patients’ healthcare outcomes [6].

A growing interest in assessing nurses’ practice environment has emerged, with factors that affect nursing organizational well‐being. Nurses can be affected by hospital traits, cultural foundations, and processes that support, uphold, and enhance the physical, mental, and social well‐being of the working environment, as well as their quality of life, which drive the dynamics of coexistence in practice environments [7]. Additionally, nurses’ bedside performance can impact patient outcomes, as they can exhibit positive and negative behaviors [8]. Nurses’ performance deteriorates in a negative practice environment characterized by excessive workloads, interpersonal problems, and dysfunctional leadership [9]. Next, nurses exhibit deviant behaviors such as noncompliance with social and hospital rules, which directly affect patient safety and the quality of nursing care [10]. Consequently, it is important to understand the practice environment where nurses’ daily tasks can be vital for patients and for all other hospital stakeholders. Worldwide, nursing is a tremendously stressful profession with high rates of job discontent and physical, mental, and emotional injuries [11]. Nurses have higher levels of stress at work than other healthcare professionals do because of their direct engagement in patient care, with over 50% reporting high levels of stress [12]. Additionally, interactions with patients who require urgent nursing care, such those who have cardiac diseases in cardiac care settings, can be highly stressful [13].

Compared with other hospital departments, the cardiac setting environment is essentially more stressful for nurses, who might neglect nursing care, which leads to patient deterioration and perilous [14]. A previous study indicated that burnout rates among nurses working in cardiac settings sharply increased between 20% and 50% [15]. The cardiac workplace, such as the cardiac care unit, is the most exhausted and complicated setting where nurses experience heavy workloads, interpersonal conflicts, and, inevitably, health‐related concerns for cardiac nurses [16]. According to a recent study in Iraq, nurses working in an unsuitable practice environment were at risk of developing health issues; hence, they expressed a low level of job performance [17]. This study aims to evaluate nurses’ perceptions of the practice environment in XXX cardiac hospitals. Additionally, this study seeks to identify nurse‐related factors that influence nurses’ nursing practices in these hospitals.

## 2. Methods

### 2.1. Study Design and Setting

This descriptive cross‐sectional study was conducted in 2024 to examine nurses’ perceptions of the practice environment at three specialized cardiac hospitals (**XX, XXX, and XXXX**) in **XY** and **YY**. These hospitals cover a wide spectrum of patients from Baghdad and neighboring cities and serve as referral specialized hospitals for cardiovascular patients in the capital of **YY**, XXX. The study followed the Strengthening the Reporting of Observational Studies in Epidemiology (STROBE) checklist for reporting cross‐sectional studies.

### 2.2. Study Participants

All nurses working in cardiac departments, including the medical and surgical cardiac ward, coronary care unit, intensive care unit, and open‐heart surgery theater, were considered to constitute the accessible population for the study. The inclusion criteria included both sexes, having experiences in cardiac nursing for at least one year, having various nursing qualifications, working different working shifts, and providing informed consent for participation. Nurses unwilling to participate and those with managerial tasks were excluded from the study.

The required sample size was estimated using the standard formula for cross‐sectional studies. The calculation was informed by parameters reported in a previous study using the Practice Environment Scale of the Nursing Work Index (PES‐NWI) [18] The estimation assumed an alpha level of 0.05, 90% statistical power, and a 95% confidence interval, with an additional 10% allowance for potential nonresponse or incomplete data. Based on these assumptions, the minimum required sample size was calculated to be 115 participants. The final sample size was calculated to be at least 115 eligible participants. Participants were recruited using a convenience sampling method from each cardiac nursing department. Departments with more nursing staff were allocated a proportionally larger share of the sample to ensure representation across units.

### 2.3. Data Collection and Measurements

Data were collected when participants were provided with the study questionnaires after their necessary consent was obtained. For those unable to complete the questionnaires for any reason, the questionnaires were completed by the principal investigator. In this study, data were collected via a constructed paper‐based self‐report questionnaire. All questionnaires were completed through participants’ self‐reported technique and took approximately 20–30 min for each nurse. The questionnaire consisted of a questionnaire concerning nurses’ sociodemographic characteristics, such as age, sex, acquired nursing qualifications, years of nursing experience, and number of training sessions in which nurses participated in the topic of managing the nursing practice environment. Data regarding nurses’ perceptions of their practice environment were collected via the Nursing Work Index (NWI) [19]. The NWI (29 items) was used to assess nurses’ perceptions of the practice environment in cardiovascular hospitals.

The nursing practice environment was measured using the PES‐NWI‐29, which includes five domains: nurse participation in hospital affairs, nursing foundations for quality of care, nurse manager ability, leadership and support of nurses, staffing and resource adequacy, and collegial nurse–physician relations. Items were measured on a 4‐point Likert scale ranging from 1 = *strongly disagree* to 4 = *strongly agree*. In the scoring system, a mean score of less than 2.5 was used to indicate a poor level of the practice environment; scores ranging from 2.5 to 3.25 were considered fair; and scores ranging from 3.26 to 4 were considered good.

The NWI‐29 questionnaire has been validated in various hospital settings, such as public hospitals [19], and has demonstrated acceptable internal consistency, with a Cronbach’s *α* coefficient = 0.97 [18].

### 2.4. Ethical Considerations

Ethical approval was obtained from the Institutional Review Board (IRB) of the College of Nursing/University of **XXX** Research Ethics Committee prior to commencing data collection (approval no. UOB.CON.23.008) on 19 September 2023, in accordance with the principles of the Declaration of Helsinki. Nurses were invited for interviews and reminded that participation was voluntary, and the completion of the survey was taken as implied consent. The participants had the right to withdraw at any time before submission with no financial compensation. No incentives were offered for participation in the study. The data were stored securely to protect the participants’ personal information and were exclusively accessible by the principal researcher.

### 2.5. Statistical Analysis

Statistical analysis was performed via IBM SPSS Statistics 26, a program that is a component of the Statistical Package for the Social Sciences. Seventeen of the 132 questionnaires were deemed incomplete because of missing data regarding nurses’ perceptions of the practice environment. The analysis did not include these questionnaires. Complete data were obtained from 115 patients. A descriptive analysis was performed for nurses’ sociodemographic and perception‐related data, which were in the form of categorical variables presented as frequencies and percentages, and the continuous variables were presented as the means and standard deviations. Multiple linear regression was subsequently performed to determine the joint effect of the sociodemographic characteristics of nurses on their level of practice environment. To meet assumptions of multiple linear regression, Pearson correlation studies were carried out to investigate the associations between the dependent and independent variables and validate the linearity assumption before conducting multiple linear regressions. Additionally, tolerance and variance inflation factor (VIF) statistics were used to evaluate multicollinearity among the independent variables. The Durbin‒Watson statistic was used to confirm the independence of errors, standardized residual analysis was used to confirm the residuals’ normality, and the residuals’ distribution was examined to confirm homoscedasticity. Additionally, normalized residual values with a threshold of ±3 were used to analyze outliers. These procedures guaranteed that the data satisfied the prerequisites for regression analysis. The level of statistical significance for the current study was set at *p* ≤ 0.05.

## 3. Results

The sociodemographic characteristics of the participating nurses revealed that female nurses were dominant in the study sample, accounting for 73% of the total nurses, and 78.3% of them were aged between 20 and 30 years, with a median age of M ± SD = 27 ± 6 years. Fewer than half (47.8%) of the nurses held a diploma degree in nursing, with 41.7% having 1‐2 years of experience working in cardiac nursing, with a mean of (M ± SD = 5.77 ± 6.44) years, and their participation in training sessions regarding nursing work management included 41.7% of them having 1‐2 sessions (Table 1).

**TABLE 1:** Sociodemographical characteristics of nurses in the cardiac hospitals (no = 115).

Nurses characteristics	Groups	Frequency	Percent (%)
1. Sex	Male	31	27
	Female	84	73

2. Age groups	20–30	90	78.3
	31–40	18	15.7
	41–50	7	6.1

*M ± SD (27 ± 6.89)*			
3. Nursing qualification	Preparatory Graduated	32	27.8
	Diploma in Nursing	55	47.8
	Bachelor in Nursing	26	22.6
	Postgraduate Qualification in Nursing	2	1.7

4. Years of Experience in Nursing	1‐2 (years)	48	41.7
	3‐4 (years)	16	13.9
	5–10 (years)	36	31.3
	> ± 10 (years)	15	13

*M ± SD (5.77 ± 6.44)*			
5. Number of training sessions	No training	33	28.7
	1‐2 sessions	48	41.7
	3‐4 sessions	22	19.1
	5–10 sessions	11	9.6
	More than 10 sessions	1	0.9

Abbreviation: M ± SD = mean ± standard deviation.

Table 2 shows that the cardiac nurses’ perceptions of their practice environment in the cardiac hospitals were good in most domains, with a total mean score of M ± SD = 3.38 ± 0.02. The mean scores of the cardiac nurses were good for their perceptions of nursing supervisor abilities, leadership, and staff support (M ± SD = 3.37 ± 0.17); their perceptions of participation in hospital committees (M ± SD = 3.35 ± 0.12); their perceptions of staffing and resource adequacy (M ± SD = 3.40 ± 0.08); and their perceptions of collegial nurse‒physician relationships (M ± SD = 3.40 ± 0.09).

**TABLE 2:** Cardiac nurses’ perceptions for their practice environment in cardiac hospitals.

Dimensions of Practice Environment Scale of the Nursing Work Index	M	SD
A. Nursing supervisor abilities, leadership, staff support	3.37	0.17
B. Nurses’ participation in hospital committees	3.35	0.12
C. Staffing and resources adequacy	3.40	0.08
D. Collegial nurse‒physician relations	3.40	0.09
Overall level of nurses’ perceptions on practice environment in the cardiac hospitals	3.38	0.02

*Note:* M = mean.

Abbreviation: SD = standard deviation.

**FIGURE 1:**
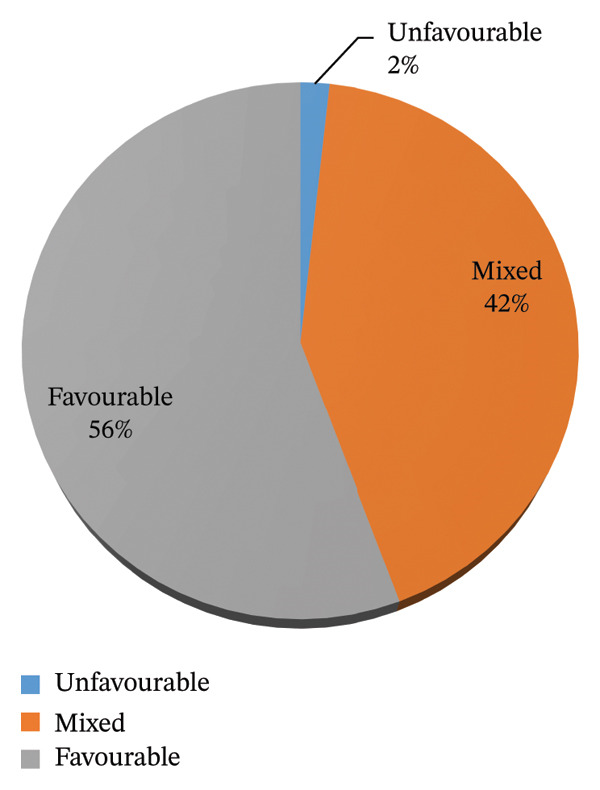
Descriptive analysis of the level of practice environment for the studied samples.

Figure 1 shows that 56% of the cardiac nurses had good practices in cardiac hospitals, whereas 42% of them had fair practices, and only 2% had poor practices.

The linear regression model (Table 3) indicates that several variables are significantly associated with nurses’ perceptions of the practice environment in cardiac hospitals (*p* value = *p* ≤ 0.014). Nurses’ qualifications are the most significant variable, with the strongest statistical evidence for influencing nurses’ perceptions, reflected by a coefficient of 0.308 (*p* = 0.002). Nurses’ years of experience in cardiac nursing follow, with a coefficient of −0.284 (*p* = 0.027), suggesting that nurses with lower levels of experience, on average, score lower than do nurses with high levels of nursing experience. Nurses’ participation in training sessions regarding nursing work management also has a relevant effect, with a coefficient of 0.216 (*p* = 0.014), indicating that while its impact is smaller, it has a significant effect on nurses’ perceptions of the practice environment in cardiac hospitals.

**TABLE 3:** Multiple linear regression models evaluating the effect of cardiac nurses’ sociodemographic characteristics and their overall practice environment in the cardiac hospitals.

Nurses sociodemographic characteristics	Unstandardized coefficients		Standardized coefficients	t	Sig.^∗^	R	F	Sig.^∗∗^ (F)
	(β)	SE	Beta					
Sex (female)	0.079	0.080	0.096	0.991	0.324	0.348	3.004	0.014^∗^
Nursing qualification (diploma)	0.150	0.048	0.308	3.151	0.002^∗^			
Age (20‒30 years)	0.006	0.077	0.010	0.080	0.936			
Years of experience in nursing (1‐2 years)	−0.094‐	0.042	−0.284‐	−2.238‐	0.027^∗^			
Number of training sessions (1‐2 sessions)	0.083	0.040	0.216	2.069	0.041^∗^			

*Note:* t value = t‐statistic, R = multiple correlation coefficient f = (F‐statistic, overall model significance), Sig.^∗^ = level of significance for *t* value, Sig.^∗∗^ = level of significance for F value (ANOVA) table of regression analysis (overall model significance).

Abbreviation: SE = standard error.

^∗^ *p* value = ≤ 0.05.

## 4. Discussion

A balanced and productive practice environment can mitigate the adverse effects of the negativity of cardiac nurses’ perceptions in the hospital setting [20]. Among the sample of cardiac nurses in a stressful hospital environment who care for critical and complex patients, the current study revealed that the perceptions of those nurses about their practice environment may vary. To the best of our knowledge, this is the first study to investigate the level of perception among cardiac nurses, including how the practice environment may differ in terms of their characteristics, which has not been previously studied. This is precisely the context of the population that could benefit from the results of this study.

According to the nurses’ characteristics in the current study, they were younger, and most were females. Similar results were reported in a recent study investigating the practices and attitudes of cardiac nurses toward the work environment in a cardiac setting, which indicated that female and young nurses were dominant [21]. In contrast, a recent Iraqi study revealed that male nurses represented the majority of the sample in terms of median age [22]. Younger, female nurses with limited training participation reported more positive perceptions of the practice environment. This may reflect recent education, early‐career enthusiasm, or limited exposure to workplace challenges. However, as this is based on self‐reported data, caution is needed in interpretation, and future qualitative studies could explore these perceptions in more depth. Our study also revealed that nearly half of the participating cardiac nurses held a diploma degree in nursing and almost the same percentage were novel nurses with few years of nursing experience in cardiac hospitals. These results are in agreement with those of a previous study in which the clinical practice of cardiac nurses revealed that less than half had diplomas or a certified course in nursing and that one‐third of them had five years of experience in cardiac care settings [23]. Furthermore, the nurses in our study poorly participated in training sessions on nursing work management. This result is identical to qualitative study results, which revealed that nurses limitedly participated in the training session because of significant learning obstacles across the training process [24]. Given that cardiac nurses were young and likely to have fair nursing education and nursing experiences, with little willingness in the training sessions, this study might surmise that these nurses had limited time and that other activities took precedence over their perceptions of the work environment.

More cardiac nurses in the current study reported a good level of perception of the work practice environment in the cardiac setting. Nurses believe that their cardiac work nursing practice environment has the capacity for optimal leadership, staff support, and nursing supervisory skills. A previous study by Siouta et al. revealed that cardiac nurses in cardiovascular care felt that healthcare professionals have authority in decision‐making [25]. A literature review revealed that working in healthier cardiac workplaces was linked to reduced 30‐day inpatient mortality rates, lower failure‐to‐rescue rates, greater postcardiac arrest survival, and fewer factors connected to missed care [26]. Furthermore, comprehensive interaction and team support for cardiac nurses provide a more thorough and targeted procedure, give nurses more time to address any care coordination concerns, clarify patients’ questions, and provide in‐depth care instruction [27]. The different interpretations of the work environment for nursing practice, each of which is context sensitive, reflect the variety of coordination, training, and abilities related to nursing jobs. Moreover, optimal nursing leadership in the environment of healthcare institutions must concentrate on creating creative programs with long‐term effects to meet the urgent demands of nurse clinicians [28]. Similarly, there is strong evidence that nursing supervision abilities play a part in focusing on nurse‐directed teams and nurses’ adherence to guidelines at cardiac hospitals [29].

The findings revealed that cardiac nurses valued nursing participation in hospital committees. This finding was in line with a Saudi survey indicating that nurses’ involvement in medical affairs has a favorable and significant effect on the quality of patient care and high job satisfaction, which reflects the effects of nurses’ job enjoyment [30]. The current study also highlights the positive view of cardiac nurses on staffing and resource adequacy. A sufficient number of nurses is essential for fortifying the healthcare system, enhancing health coverage, and accomplishing all health goals [31]. However, the World Health Organization reported that by 2035, there will be a need for 12.9 million nurses and that there will be a deficit of 7.2 million health workers to provide healthcare services globally [32].

This study also revealed that nurses had positive perceptions of collegial nurse–physician relations. This result is congruent with a survey from the United States that revealed a positive relationship in the workplace between collegiality and the nurse‒physician relationship, which in turn increased job satisfaction [33]. This is attributed to the favorable relationship between nursing foundations and collegial nurse‒physician relationships, which are beneficial because they increase work engagement and the quality of care and reduce quiet quitting [34]. Nurses’ practice behaviors can be influenced by their workplace, which can affect the standard of healthcare services and the effectiveness of healthcare institutions.

Additionally, multiple linear regression revealed a significant relationship between nurses’ perceptions of the practice environment working in cardiac hospitals and their qualifications, years of experience in cardiac nursing, and participation in training sessions regarding nursing work management. This finding mirrors Egyptian study results, which revealed a highly statistically significant relationship between nurses’ perceptions of the work practice environment and their sociodemographic factors (*p* ≤ 0.001) [35]. For example, certified perianesthesia nurses who regularly attended continuous hospital educational programs had significantly better assessments of their workload and intention to stay [36]. The establishment of the optimal nursing practice environment depends on a cooperative development process, a strong infrastructure of resources, and well‐defined roles for the clinical care team. The intricate nature of contemporary cardiovascular nursing care necessitates skilled, specialized nurses to guarantee superior patient results. The environments of specific cardiac settings and the characteristics of the nurse population are closely related. In actuality, the complexity of medical management and advanced nursing care can be overcome by the availability of support staff (leaders), support staff teams, and realistic workload assignments.

## 5. Limitations and Implications

To the best of our knowledge, this is the first study to investigate nurses’ perceptions of the practice environment in **XXX** cardiac hospitals. This study used standard instruments, which improved the validity of the findings and interpretation. However, it is crucial to consider that the data collected for this study were based on participant perceptions and should be interpreted with caution. Additionally, the study participants were chosen from cardiovascular hospitals; therefore, the findings might not be applicable to other healthcare settings. Future research should extend to nurses in diverse healthcare organizations and consider using mixed‐methods or longitudinal designs to further explore and validate these perceptions, including factors influencing differences by age and gender. Finally, because the data were collected using self‐reported questionnaires, responses may be subject to social desirability or response bias.

## 6. Conclusion

This first practice environment evaluation has revealed a variety of perspectives regarding cardiac nurses’ perceptions that future nursing authorities might need. The study revealed that cardiac nurses held positive perceptions of their nursing practices in cardiac care settings. This is particularly true in terms of leadership, staff support, nursing supervisory skills, participation in hospital committees, staffing and resource adequacy, and collegial nurse‒physician relations. Nurses’ perceptions are a controllable contextual component by which nursing practice environments should focus on strengthening the relational competences of cardiac healthcare facilities.

**Nomenclature**
NWI: Nursing Work Index

## Author Contributions

Ali Hussein Alek Al‐Ganmi: conceptualization, methodology, formal analysis, data curation, writing‒original draft, writing‒review and editing, and project administration. Tahseen Rajab Mohammed: methodology, validation, investigation, Resources, data curation, writing‒original draft, and project administration. Ayad Majid Mousa: conceptualization, formal analysis, investigation, resources, and data curation. Haider Mohammed Majeed: conceptualization, methodology, formal analysis, investigation, and project administration. Abdulellah M. Alsolais: writing‒original draft and writing‒review and editing.

## Funding

No funding was received for this manuscript.

## Ethics Statement

Ethical approval for this study was obtained from the Institutional Review Board of College of Nursing/University of Baghdad Research Ethics Committee (IRB: UOB.CON.23.008), aligning with the Declaration of Helsinki principles as outlined by the World Medical Association (https://www.wma.net/policies-post/wma-declaration-of-helsinki/). Written informed consent was obtained from all nurses prior to their participation, reminder them that participation is voluntary and the completion of the survey was taken as implied consent. Participants had the right to withdraw at any time before submission with no financial compensation.

## Consent

Please see the Ethics Statement.

## Conflicts of Interest

The authors declare no conflicts of interest.

## Data Availability

The data that support the findings of this study are available from the corresponding author, but restrictions apply to the availability of these data, which were used under license for the current study and are not publicly available. However, the data can be obtained from the corresponding author upon reasonable request and with appropriate permissions.

## References

[1] Sabri K. S. and Hassan H. S. , An Effectiveness of Nurse’s Knowledge and Practices Program Toward Care of Patient Undergoing Cardiopulmonary Bypass, Iraqi National Journal of Nursing Specialties. (2023) 36, no. 1, 10–16.

[2] Jakimowicz S. , Perry L. , and Lewis J. , Insights on Compassion and Patient-Centred Nursing in Intensive Care: A Constructivist Grounded Theory, Journal of Clinical Nursing. (2018) 27, no. 7-8, 1599–1611, 10.1111/jocn.14231, 2-s2.0-85041675244. 29266484

[3] Guttormson J. L. , Calkins K. , McAndrew N. , Fitzgerald J. , Losurdo H. , and Loonsfoot D. , Critical Care Nurse Burnout, Moral Distress, and Mental Health During the COVID-19 Pandemic: A United States Survey, Heart & Lung. (2022) 55, 127–133, 10.1016/j.hrtlng.2022.04.015. 35561589 PMC9050623

[4] MacKay S. C. , Smith A. , Kyle R. , and Beattie M. , What Influences Nurses’ Decisions to Work in Rural and Remote Settings? A Systematic Review and Meta-Synthesis of Qualitative Research, Rural and Remote Health. (2021) 21, no. 1, 10.22605/rrh6335. 33653078

[5] Lalonde M. et al., Part 2: New Graduate Nurse Transition Into the Intensive Care Unit: Summative Insights From a Longitudinal Mixed-Methods Study, Research and Theory for Nursing Practice. (2021) . 10.1891/RTNP-D-21-00014 34518357

[6] Baiez Y. K. and Mohammod W. K. , Evaluation of Nursing staff Performance in Cardiac Care Units at Teaching and Non Teaching Hospitals in Kirkuk City: A Comparative Study, Iraqi National Journal of Nursing Specialties. (2015) 28, no. 1, 77–85.

[7] Avallone F. and Bonaretti M. , Organizational Well-Being. to Improve Job Quality in Publish Administrations, 2003, Soveria Mannelli.

[8] Alsadaan N. , Ramadan O. M. E. , and Alqahtani M. , From Incivility to Outcomes: Tracing the Effects of Nursing Incivility on Nurse Well-Being, Patient Engagement, and Health Outcomes, BMC Nursing. (2024) 23, no. 1, 10.1186/s12912-024-01996-9. PMC11092052 38741096

[9] El-Sayed A. A. I. et al., The Effect of Organizational Silence on the Relationship Between Workplace Ostracism and Nurses’ Procrastination Behavior: A Structural Equation Modeling, International Nursing Review. 10.1111/inr.13050 39367864

[10] Mansor M. , Ibrahim R. M. , Afthanorhan A. , and Salleh A. M. M. , The Mechanism of Anger and Negative Affectivity on the Occurrence of Deviant Workplace Behavior: An Empirical Evidence Among Malaysian Nurses in Public Hospitals, Belitung Nursing Journal. (2022) 8, no. 2, 115–123, 10.33546/bnj.1994. 37521891 PMC10386817

[11] Babapour A.-R. , Gahassab-Mozaffari N. , and Fathnezhad-Kazemi A. , Nurses’ Job Stress and Its Impact on Quality of Life and Caring Behaviors: A Cross-Sectional Study, BMC Nursing. (2022) 21, no. 1, 10.1186/s12912-022-00852-y. PMC8968092 35361204

[12] Vahedian-Azimi A. , Hajiesmaeili M. , Kangasniemi M. et al., Effects of Stress on Critical Care Nurses: A National Cross-Sectional Study, Journal of Intensive Care Medicine. (2019) 34, no. 4, 311–322, 10.1177/0885066617696853, 2-s2.0-85033678268. 29277137

[13] Goudarzian A. H. , Nikbakht Nasrabadi A. , Sharif-Nia H. , Farhadi B. , and Navab E. , Exploring the Concept and Management Strategies of Caring Stress Among Clinical Nurses: A Scoping Review, Frontiers in Psychiatry. (2024) 15, 10.3389/fpsyt.2024.1337938. PMC11165118 38863606

[14] Isam S. R. and Hassan H. S. , Effectiveness of Cardiac Rehabilitation Instructional Program on Health-Related Quality of Life for Patients Undergone Coronary Artery Bypass Graft Surgery, Iraqi National Journal of Nursing Specialties. (2023) 36, no. 1, 59–70, 10.58897/injns.v36i1.809.

[15] Howie-Esquivel J. , Byon H. D. , Lewis C. , Travis A. , and Cavanagh C. , Quality of Work-Life Among Advanced Practice Nurses who Manage Care for Patients With Heart Failure: The Effect of Resilience During the Covid-19 Pandemic, Heart & Lung. (2022) 55, 34–41, 10.1016/j.hrtlng.2022.04.005. 35447467 PMC8995301

[16] Kim J. A. , Hwang W. J. , and Jin J. , An Exploration of Contextual Aspects That Influence Cardiovascular Disease Risks Perceived by Workers in a Small–Medium-Sized Workplace, International Journal of Environmental Research and Public Health. (2020) 17, no. 14, 10.3390/ijerph17145155. PMC7399942 32708886

[17] Kadhim H. and Qassem W. J. , Impact of Physical Work Environment Upon Nurses’ Job Performance in Al-Nassiryah City Hospitals, Iraqi National Journal of Nursing Specialties. (2023) 36, no. 1, 16–25, 10.58897/injns.v36i1.707.

[18] Juanamasta I. G. , Aungsuroch Y. , Fisher M. L. , Nuryani S. N. A. , and Ayuningsih N. N. , Translation and Validation Study of the Indonesian Version of the Practice Environment Scale of the Nursing Work Index, International Journal of Nursing Science. (2023) 10, no. 4, 511–517, 10.1016/j.ijnss.2023.09.018. PMC10667313 38020847

[19] Lake E. T. , Development of the Practice Environment Scale of the Nursing Work Index, Research in Nursing & Health. (2002) 25, no. 3, 176–188, 10.1002/nur.10032, 2-s2.0-0036615403. 12015780

[20] Labrague L. J. , The Impact of Job Burnout on Nurses’ Caring Behaviors: Exploring the Mediating Role of Work Engagement and Job Motivation, International Nursing Review. (2024) 71, no. 3, 653–660, 10.1111/inr.12899. 37908133

[21] Zhao E. , Lowres N. , Bloomfield J. , Weddell J. , Tofler G. , and Gallagher R. , Current Practices and Attitudes of Cardiac Nurses Regarding Cognitive Screening in Patients With Acute Coronary Syndrome, Heart Lung & Circulation. (2024) 33, no. 7, 1050–1057, 10.1016/j.hlc.2024.01.017. 38462415

[22] Majeed H. M. , Hassan A. F. , and Al-Ganmi A. H. A. , Nurses’ Performance and Perceived Barriers Regarding Pressure Ulcers’ Prevention for Critically Ill Patients at Baghdad Teaching Hospitals, Journal of Nature and Science of Medicine. (2024) 7, no. 4, 293–298, 10.4103/jnsm.jnsm_87_24.

[23] Wasfi H. and Ajil Z. W. , The Level of Nurses Knowledge of Discharge Planning for Pediatric Congenital Heart Disease, Iraqi National Journal of Nursing Specialties. (2025) 38, no. 1, 79–88.

[24] Bae M.-J. and Shin N.-M. , Factors Impeding Learning at Various Stages of Simulation Training as Experienced by Nursing Students, Nursing Forum. (2024) 2024, no. 1, 10.1155/2024/6808399.

[25] Siouta E. , Olsson U. , and Waldréus N. , Nurses’ Perceptions of Patient Involvement in Shared Decision-Making in Cardiovascular Care, Heliyon. (2023) 9, no. 12, 10.1016/j.heliyon.2023.e22890. PMC10746438 38144325

[26] Vozzella G. M. and Hehman M. C. , Cardiovascular Nursing Workforce Challenges: Transforming the Model of Care for the Future, Methodist DeBakey Cardiovascular Journal. (2023) 19, no. 2, 90–99, 10.14797/mdcvj.1188. 36910553 PMC10000318

[27] Vaismoradi M. , Rae J. , Turunen H. , and Logan P. A. , Specialized Nurses’ Role in Ensuring Patient Safety Within the Context of Telehealth in Home Care: A Scoping Review, Digital Health. (2024) 10, 10.1177/20552076241287272. PMC11459674 39381815

[28] Reis da Silva T. M. H. , Sedky A. , Navigating Healthcare Complexity: Integrating Business Fundamentals Into Nursing Leadership, Resiliency Strategies for Long-Term Business Success, 2025, IGI Global, Hershey, PA, 145–168.

[29] Hayman L. L. , Berra K. , Fletcher B. J. , and Houston Miller N. , The Role of Nurses in Promoting Cardiovascular Health Worldwide, Journal of the American College of Cardiology. (2015) 66, no. 7, 864–866, 10.1016/j.jacc.2015.06.1319, 2-s2.0-84939560269. 26271070

[30] Alqasmi I. and Ahmed S. , Mediating Role of Nurse Job Enjoyment and Participation in Medical Affairs on Quality of Patient Care in Saudi Hospitals, TQM Journal. (2025) 37, no. 1, 106–124, 10.1108/tqm-04-2023-0119.

[31] van Kraaij J. , de Vries N. , Wessel H. et al., Enhancing Work Environments and Reducing Turnover Intention: A Multicenter Longitudinal Cohort Study on Differentiated Nursing Practices in Dutch Hospitals, BMC Nursing. (2025) 24, no. 1, 10.1186/s12912-024-02681-7. PMC11721496 39794770

[32] Adams R. , Ryan T. , and Wood E. , Understanding the Factors That Affect Retention Within the Mental Health Nursing Workforce: A Systematic Review and Thematic Synthesis, International Journal of Mental Health Nursing. (2021) 30, no. 6, 1476–1497, 10.1111/inm.12904. 34184394

[33] Davis S. G. , Davis E. , Kintz K. , and Opsahl A. , Nurse Educator Perceptions of Workplace Collegiality, Nurse Educator. (2022) 47, no. 5, 288–292, 10.1097/nne.0000000000001194. 35324495

[34] Moisoglou I. , Katsiroumpa A. , Katsapi A. , Konstantakopoulou O. , and Galanis P. , Poor Nurses’ Work Environment Increases Quiet Quitting and Reduces Work Engagement: A Cross-Sectional Study in Greece, Nursing Reports. (2025) 15, no. 1, 10.3390/nursrep15010019. PMC11767771 39852641

[35] M Hegazy A. , Ibrahim M. M. , A Shokry W. , and A El shrief H. , Nurses’ Perception of Work Environment Factors and Its Relation With Their Work Engagement, Egyptian Journal of Health Care. (2022) 13, no. 1, 280–293, 10.21608/ejhc.2022.216639.

[36] Ross J. , Organizational Support, Workload, and Intent to Stay: Work Environment Perceptions in Perianesthesia Nursing Units, Journal of PeriAnesthesia Nursing. (2017) 32, no. 4, 287–294, 10.1016/j.jopan.2015.07.001, 2-s2.0-85008656027. 28739060

